# Comparative accuracy of two commercial AI algorithms for musculoskeletal trauma detection in emergency radiographs

**DOI:** 10.1007/s10140-025-02353-2

**Published:** 2025-06-09

**Authors:** Jarno T. Huhtanen, Mikko Nyman, Roberto Blanco Sequeiros, Seppo K. Koskinen, Tomi K. Pudas, Sami Kajander, Pekka Niemi, Hannu J. Aronen, Jussi Hirvonen

**Affiliations:** 1 Faculty of Health and Well-being, Turku University of Applied Sciences, Joukahaisenkatu, Turku, 20520 Finland; 2 Department of Radiology, University of Turku, Turku, Finland; 3 Department of Radiology, University of Turku, Turku University Hospital, Turku, Finland; 4 Terveystalo Inc, Jaakonkatu 3, Helsinki, Finland; 5 Department of Radiology, Faculty of Medicine and Health Technology, Tampere University, Tampere University Hospital, Tampere, Finland

**Keywords:** Artificial intelligence, Fracture, Radiograph, Appendicular skeleton

## Abstract

**Purpose:**

Missed fractures are the primary cause of interpretation errors in emergency radiology, and artificial intelligence has recently shown great promise in radiograph interpretation. This study compared the diagnostic performance of two AI algorithms, BoneView and RBfracture, in detecting traumatic abnormalities (fractures and dislocations) in MSK radiographs.

**Methods:**

AI algorithms analyzed 998 radiographs (585 normal, 413 abnormal), against the consensus of two MSK specialists. Sensitivity, specificity, positive predictive value (PPV), negative predictive value (NPV), accuracy, and interobserver agreement (Cohen’s Kappa) were calculated. 95% confidence intervals (CI) assessed robustness, and McNemar’s tests compared sensitivity and specificity between the AI algorithms.

**Results:**

BoneView demonstrated a sensitivity of 0.893 (95% CI: 0.860–0.920), specificity of 0.885 (95% CI: 0.857–0.909), PPV of 0.846, NPV of 0.922, and accuracy of 0.889. RBfracture demonstrated a sensitivity of 0.872 (95% CI: 0.836–0.901), specificity of 0.892 (95% CI: 0.865–0.915), PPV of 0.851, NPV of 0.908, and accuracy of 0.884. No statistically significant differences were found in sensitivity (*p* = 0.151) or specificity (*p* = 0.708). Kappa was 0.81 (95% CI: 0.77–0.84), indicating almost perfect agreement between the two AI algorithms. Performance was similar in adults and children. Both AI algorithms struggled more with subtle abnormalities, which constituted 66% and 70% of false negatives but only 20% and 18% of true positives for the two AI algorithms, respectively (*p* < 0.001).

**Conclusions:**

BoneView and RBfracture exhibited high diagnostic performance and almost perfect agreement, with consistent results across adults and children, highlighting the potential of AI in emergency radiograph interpretation.

**Supplementary Information:**

The online version contains supplementary material available at 10.1007/s10140-025-02353-2.

## Graphical abstract

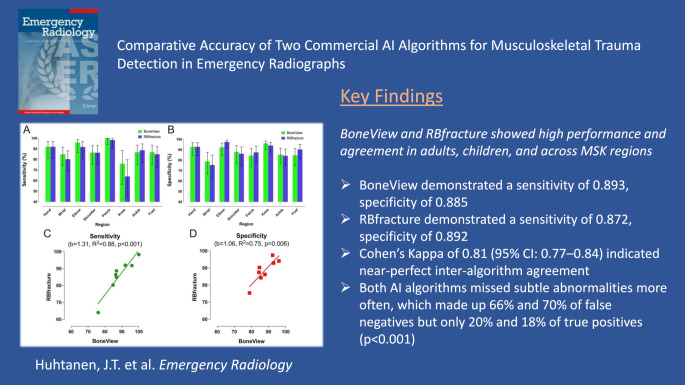

## Introduction

Despite the rapid development of imaging modalities in radiology, radiographs remain essential in evaluating trauma findings. Indeed, missed fractures are the leading cause of interpretation errors [1]. Artificial intelligence (AI), especially when combined with human assessment, can reduce these errors [2]. Technical advancements in deep learning (DL), especially convolutional neural networks (CNN) [3], have sparked great promise in radiograph interpretation [4, 5], with 777 FDA-approved AI solutions targeting this field [6]. However, AI has not consistently reduced clinically significant discrepancies [7]. AI effectively detects radiographic abnormalities in various musculoskeletal (MSK) regions, such as the upper [8–11] and lower extremities [12, 13]. AI stand-alone performances can achieve and surpass the level of a subspecialized radiologist [14], detecting findings that radiologists are missing [15]. In addition, AI has been shown to decrease the number of interpretation errors in various age groups [16].

Yet, AI can still be prone to errors in radiograph interpretation [10, 15, 17–25]. Many promising results are usually from one abnormality detection in a single location [26], and studies including multiple MSK regions are rare [14, 27, 28]. In addition, few studies have examined AI performance in pediatric patients, with most existing studies focusing on specific regions, such as the elbow [29]. This is clinically relevant, given that pediatric radiographs are more challenging than those from adults [30]. Thus, there is a clear need for independent studies comparing commercially available AI algorithms for trauma detection from radiographs, including data from multiple imaging devices, multiple MSK regions, and a wide patient age range.

The current study sought to examine and compare the performance of two commercially available AI algorithms for trauma radiograph interpretation against the ground truth set by two MSK specialists. To address the knowledge gaps in this field, we included data from (a) adults and children; (b) multiple MSK regions, thus simulating a realistic clinical scenario yet having a sufficient number of cases in each category to provide meaningful comparisons; and (c) multiple imaging devices to address generalizability. We hypothesized that (1) the two AI algorithms have comparable overall performances and (2) the AI performances are comparable across MSK regions and in different age groups.

## Materials and methods

### Study ethics

This retrospective study received ethical approval from the Ethics Committee of the University of Turku (ETMK Dnro: 38/1801/2020). The research complied with the Declaration of Helsinki, and due to its retrospective nature, the need for informed consent was waived by the Ethics Committee of the Hospital District of Southwest Finland.

### Study population and design

This retrospective study reviewed the diagnostic performance of two AI algorithms (BoneView and RBfracture) in detecting abnormalities on emergency MSK radiographs. The dataset included 998 radiographs using purposive sampling collected between 2018 and 2021, all of which were annotated at the case level by two MSK specialists with 20 and 25 years of experience; their consensus (normal/abnormal) was used as the ground truth. The abnormal cases encompassed all trauma-related fractures and dislocations as determined by the ground truth without further subclassification in acute vs. chronic or displaced vs. nondisplaced findings. Normal cases were defined as the absence of traumatic findings, without documentation of possible hardware, degenerative disease, or osteoporosis. As predetermined in the study design, the two radiologists conducted a joint consensus read of all cases. The use of conventional radiographs for reference standard and the use of consensus reporting can be seen as common practice in many previous AI fracture detection studies [2, 10], representing the standard of care for diagnosing fractures and dislocations. The two radiologists classified abnormal findings as being either obvious or subtle based on their subjective assessments and a joint consensus. In addition, we did not systematically re-review the false positive predictions by the AI algorithms for errors in the reference standard. Possible chronic findings were treated as false positives. All radiographs were evaluated in a picture archiving and communication system (PACS) using diagnostic monitors and realistic reading conditions.

Various MSK regions were included (shoulder, elbow, wrist, hand, pelvis and hip, knee, ankle and foot), number of cases varying from 117 in the shoulder to 136 in the ankle. In addition, sampling was stratified by MSK region, rate of normal vs. abnormal cases, and age group. Radiographs from multiple imaging devices were included. Radiographs from multiple regions in the same patient were allowed and treated as independent instances. Inclusion criteria were: (a) trauma indication, (b) availability of an original radiology report from either a radiology specialist or resident, and (c) primary radiographs. Exclusion criteria included (a) projections not compatible with the AI algorithms, and (b) follow-up studies. Patient demographics are presented in Table 1.

**Table 1:** Patient demographics

Demographic	All patients	Children 2–17 years	Adults ≥ 18 years
Total	998	195	803
Male	439	95	344
Female	559	100	459
Mean Age (SD)	45.2 (27.1)	12.2 (3.5)	53.6 (24.0)
Range	2–99	2–17	18–99

*SD* standard deviation

### AI algorithms used in this study

Two commercially available AI algorithms were evaluated: BoneView (Gleamer, Paris, France, v.2.5.1) and RBfracture (Radiobotics ApS, Copenhagen, Denmark, v.2.2.1), both designed for automated fracture and dislocation detection from radiographs. The AI algorithms were analyzed on the Collective Minds Radiology platform (Collective Minds Radiology, Stockholm, Sweden), a cloud-based system facilitating AI integration with radiographic data. Cases were anonymized while retaining data compatible with AI software requirements, including patient age, and uploaded to the platform in DICOM format without preprocessing. Both BoneView and RBfracture independently analyzed the images, generated binary outputs (fracture/dislocation present or absent), and, where applicable, annotated abnormalities. Annotations (bounding boxes) were not used in the analysis. In addition, any suspicious or doubtful findings from the AI algorithms were interpreted as abnormal. Data were processed in March 2025, ensuring compatibility with the platform’s latest software version. The AI algorithm outputs were recorded without knowledge of the ground truth or original reports to prevent bias.

### Statistical analyses

AI algorithm predictions were compared against the ground truth used in this study to calculate and compare diagnostic accuracy metrics. Data were expressed in terms of true positives (TP), true negatives (TN), false positives (FP), and false negatives (FN), and diagnostic accuracy metrics were calculated as sensitivity (TP/[TP + FN]), specificity (TN/[TN + FP)], PPV (TP/[TP + FP]), NPV (TN/[TN + FN]), and accuracy (true predictions divided by all predictions). We also calculated the combined diagnostic accuracy of both algorithms by assigning the case as abnormal if both or either algorithm yielded a positive prediction. Analyses were stratified according to MSK regions and age group (adults vs. children). In addition, the proportions of subtle vs. obvious cases were examined for both AI algorithms in the TP and FN groups. 95% confidence intervals (95%CIs) were determined using the Wilson score method. McNemar’s test examined differences for paired binary outcomes, applied separately to positive (*n* = 413) and negative (*n* = 585) cases to assess differences in sensitivity and specificity, respectively. Interobserver agreement between the two AI algorithms was evaluated using Cohen’s unweighted Kappa, based on a 2 × 2 contingency table of their paired predictions. Kappa was calculated in R (version 4.4.0; R Foundation for Statistical Computing, Vienna, Austria) using the psych package, with 95%CIs provided to assess the precision of the agreement estimate. Categorical variables were summarized with counts and percentages, and associations were evaluated using chi-squared tests or Fisher’s exact test where appropriate. *P* values smaller than 0.05 (two-tailed) were considered statistically significant. All statistical analyses were performed in R with the packages binom, stats, pROC, caret, and psych.

## Results

### AI algorithm performance

**Fig. 1:**
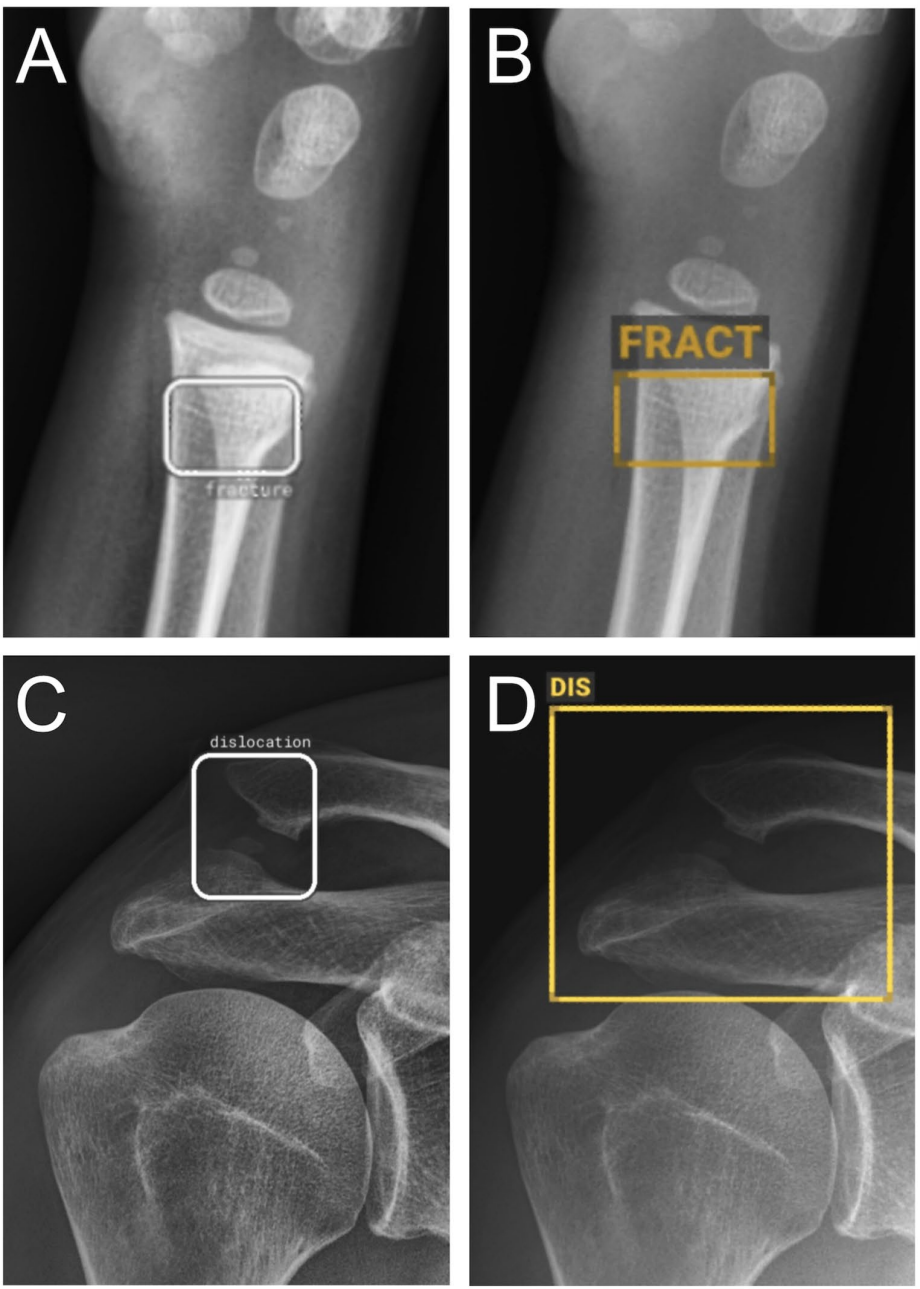
Examples of true positive fracture (top row) and dislocation (bottom row). Lateral radiograph of a 5-year-old patient with distal radius fracture classified as obvious; both RBfracture (**A**) and BoneView (**B**) correctly localized the fracture. An adult patient with an AC-joint dislocation was correctly detected by RBfracture (**C**) and BoneView (**D**)

**Fig. 2:**
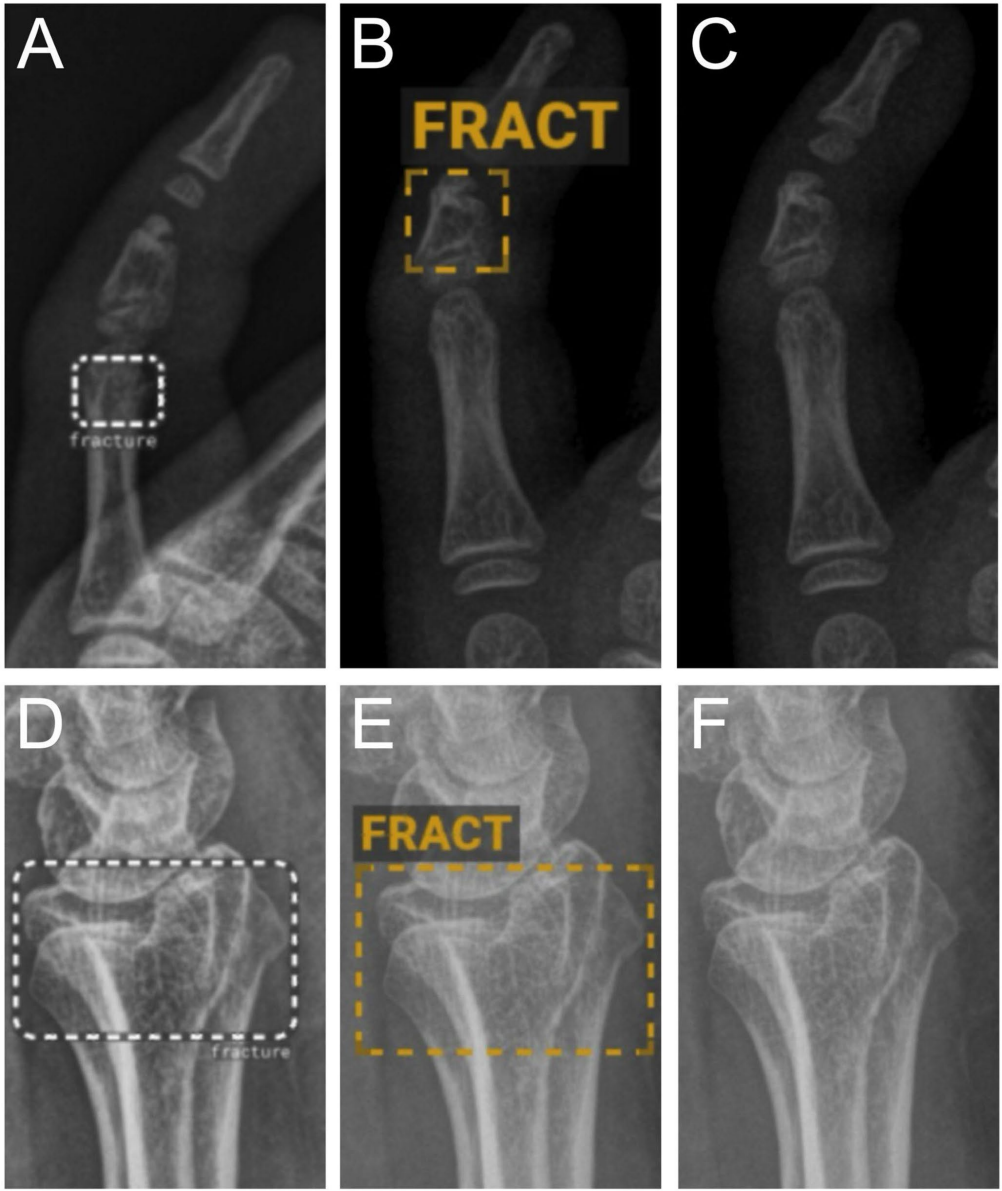
Examples of false positive (FP) findings from both AI algorithms, RBfracture (**A**, **D**) and BoneView (**B**, **E**), while images **C** and **F** show non-annotated radiographs. Of note, **A** and **B** represent FP findings in different projections between the AI algorithms. Images **A**-**C** correspond to a pediatric patient with no traumatic findings, while images **D**-**F** correspond to an adult patient with no traumatic findings in the distal radius

**Fig. 3:**
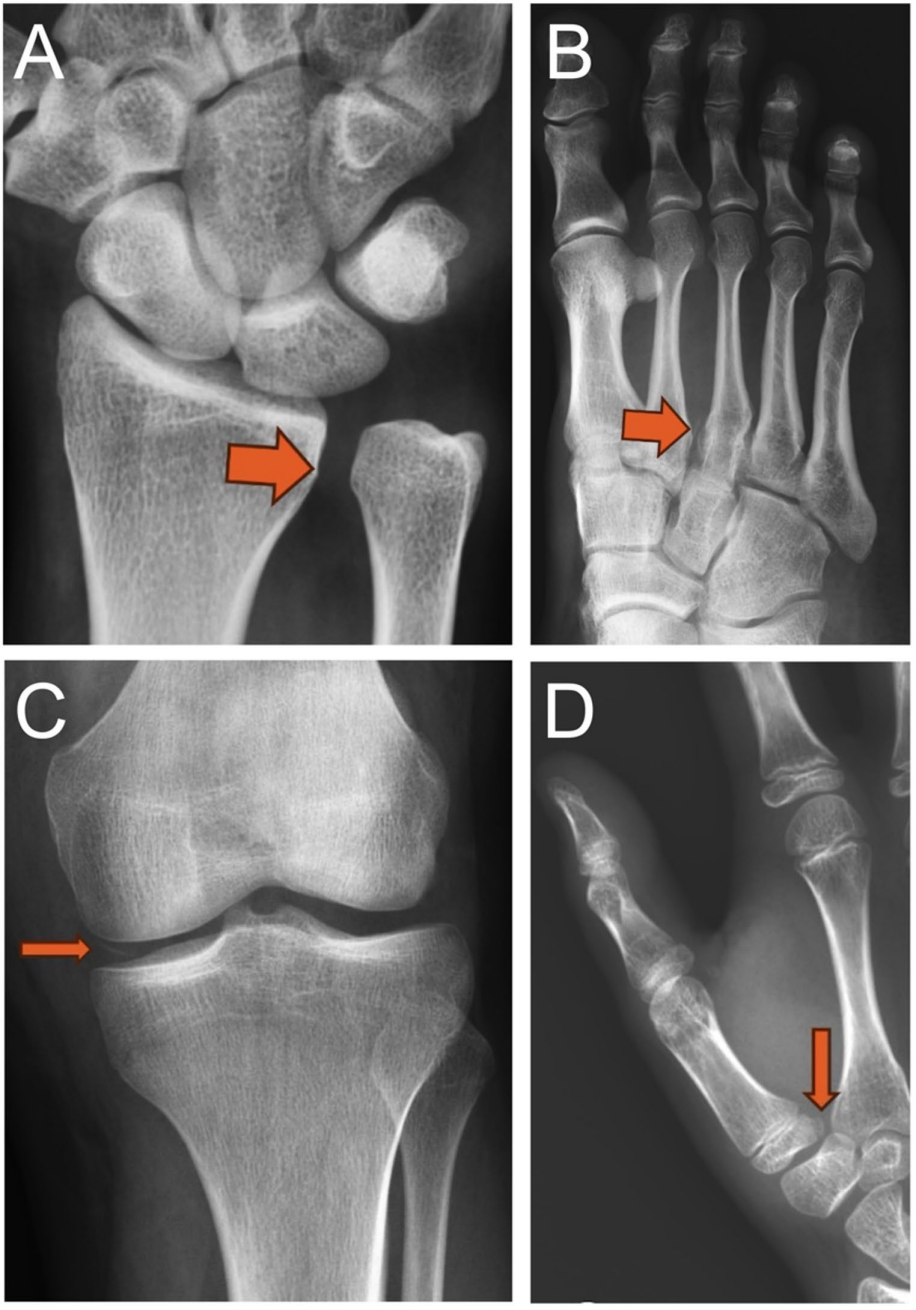
False negative (FN) findings from both AI algorithms. (**A**) DRUJ widening (arrow in **A**) in a patient with wrist trauma. (**B**) Midfoot trauma with an associated avulsion fragment between the second and third metatarsals (arrow in **B**). Both findings were classified as obvious. (**C**) Cortical fragment in the medial tibiofemoral joint after knee trauma (arrow in **C**) and (**D**) proximal first metacarpal fracture in a patient with hand trauma (arrow in **D**). Both findings were classified as subtle

Of the 998 included radiographs, 585 (59%) were classified as normal and 413 (41%) as abnormal (fracture or dislocation) as the ground truth. BoneView demonstrated a sensitivity of 0.893 (95% CI: 0.860–0.920), specificity of 0.885 (95% CI: 0.857–0.909), PPV of 0.846 (95% CI: 0.809–0.877), NPV of 0.922 (95% CI: 0.897–0.941), and accuracy of 0.889 (95% CI: 0.868–0.907). RBfracture demonstrated a sensitivity of 0.872 (95% CI: 0.836–0.901), specificity of 0.892 (95% CI: 0.865–0.915), PPV of 0.851 (95% CI: 0.814–0.882), NPV of 0.908 (95% CI: 0.881–0.929), and accuracy of 0.884 (95% CI: 0.862–0.902) (Table 2). No statistically significant differences were observed in either sensitivity (*p* = 0.151) or specificity (*p* = 0.708). Kappa was 0.81 (95% CI: 0.77–0.84), indicating almost perfect agreement between the two AI algorithms. Examples of TP, FP, and FN for BoneView and RBfracture are shown in Figs. 1 and 2, and 3.

**Table 2:** Algorithm performance metrics for both AI algorithms

	TP	TN	FP	FN	Sensitivity	Specificity	PPV	NPV	Accuracy	McNemar’s *p* (Sens)	McNemar’s *p* (Spec)	Cohen’s Kappa
BoneView (95% CI)	369	518	67	44	0.893 (0.860–0.920)	0.885 (0.857–0.909)	0.846 (0.809–0.877)	0.922 (0.897–0.941)	0.889 (0.868–0.907)	0.151	0.708	0.81 (0.77–0.84)
RBfracture (95% CI)	360	522	63	53	0.872 (0.836–0.901)	0.892 (0.865–0.915)	0.851 (0.814–0.882)	0.908 (0.881–0.929)	0.884 (0.862–0.902)			

*TP* true positive, *TN* true negative, *FP* false positive, *FN* false negative, *PPV* positive predictive value, *NPV* negative predictive value, *Sens* sensitivity, *Spec* specificity, *CI* confidence interval

Both AI algorithms were less effective in detecting subtle abnormalities compared to obvious ones. Subtle findings represented 20% of TP for BoneView and 18% for RBfracture, but respective numbers were 66% and 70% in FN (missed abnormalities), with a statistically significant difference (*p* < 0.001). Put another way, BoneView missed 28% and RBfracture missed 36% of subtle findings, but both only 5% of obvious findings. The ratio of subtle to obvious findings in missed abnormalities was not statistically significantly different between AI algorithms (χ² = 0.168, *p* = 0.682).

### Comparison of AI algorithms in different age groups

AI algorithm performance was compared between children (*n* = 195, aged 2–17 years) and adults (*n* = 803, 18–99 years) for both AI algorithms (Online resource 1). For BoneView, comparisons between adults and children showed no significant differences in sensitivity (*p* = 0.195), specificity (*p* = 0.832), NPV (*p* = 0.641), or accuracy (*p* = 0.557), though PPV was marginally lower in children (*p* = 0.053). For RBfracture, no significant differences were found in sensitivity (*p* = 0.279), specificity (*p* = 0.181), NPV (*p* = 0.533), or accuracy (*p* = 0.115), but PPV was significantly lower in children (*p* = 0.001). Inter-algorithm agreement was strong, with Kappa of 0.81 (95% CI: 0.77–0.85, almost perfect) for adults and 0.78 (95% CI: 0.69–0.87, substantial) for children.

### Comparison of AI algorithms in different MSK regions

Performance for both AI algorithms was further compared in different MSK regions (Online resource 2, Fig. 4). McNemar’s tests comparing BoneView and RBfracture indicated no significant differences in sensitivity or specificity in any of the included MSK regions (all *p* > 0.05). Kappa values ranged from 0.71 (95% CI: 0.58–0.84, Shoulder) to 0.87 (95% CI: 0.78–0.96, Elbow), indicating substantial to almost perfect agreement. Both AI algorithms tended to have the highest sensitivity in the pelvis and the lowest in the knee, and highest specificities in the elbow and the knee, and the lowest in the wrist.

**Fig. 4:**
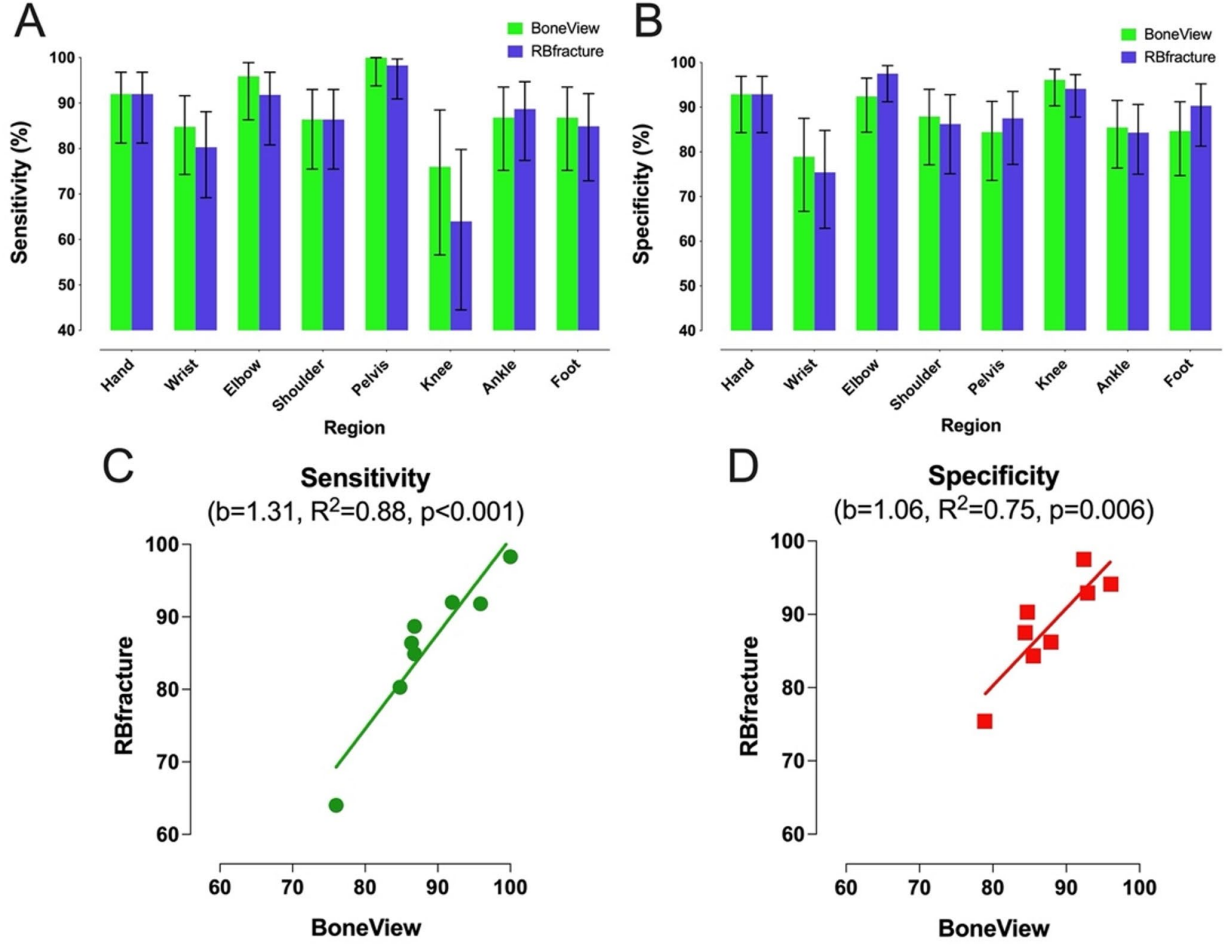
Sensitivity (**A**) and specificity (**B**) for the two AI algorithms in different MSK regions. Bars represent the estimate and error bars the 95%CIs. Correlation between diagnostic accuracy metrics in sensitivity (**C**) and specificity (**D**) from both AI algorithms in different MSK regions. Each point represents a single region

### Combined AI performance

The combined performance of both AI algorithms yielded a sensitivity of 0.920 (95% CI: 0.890–0.943), specificity of 0.834 (95% CI: 0.802–0.943), PPV of 0.797 (95% CI: 0.758–0.830), NPV of 0.937 (95% CI: 0.912–0.955) and accuracy of 0.870 (95% CI: 0.847–0.889), indicating higher detection but increased false positives combined to individual algorithms.

## Discussion

The purpose of this study was to compare the diagnostic performance of two commercially available AI algorithms, BoneView and RBfracture, in detecting acute trauma from MSK radiographs. BoneView demonstrated a sensitivity of 0.893, specificity of 0.885, and accuracy of 0.889, whereas RBfracture demonstrated a sensitivity of 0.872, specificity of 0.892, and accuracy of 0.884. There were no significant differences between AI algorithms, and there was almost a perfect agreement between them. These findings suggest acceptable diagnostic accuracy across multiple MSK regions and age groups and high consistency between AI algorithms, highlighting the potential benefit of using AI in acute trauma detection in emergency radiographs.

Both commercially available AI algorithms demonstrated high sensitivity and specificity in our dataset, including pediatric and adult patients and all relevant MSK regions. Our results are closely aligned with a recent meta-analysis reporting a pooled sensitivity of 0.91 (0.86–0.94) and a specificity of 0.89 (0.86–0.92) in fracture detection [2]. BoneView and RBfracture, with sensitivities of 0.893 and 0.872 and specificities of 0.885 and 0.892, respectively, remain within expected and acceptable performance for a stand-alone AI. In addition, our results are reasonably closely aligned with those from another meta-analysis showing AI sensitivity and specificity from external validations of 0.91 and 0.91, respectively [31]. BoneView is one of the most studied AI algorithms, and our study showed performance comparable with previous studies [2]. Both AI algorithms performed comparably to reported human ranges [32]. The combined performance of the two AI algorithms increased sensitivity but decreased specificity compared to the individual algorithms. Although deploying two algorithms for the same task may not be clinically reasonable, this approach might maximize sensitivity.

In our study, both AI algorithms tended to give more false positive predictions than false negatives, consistent with previous studies [15, 33, 34]. Although comparing the number of FP between different studies is challenging due to inherent variability in study designs, the amount of FP in our study is similar to those previously reported [15]. In addition, AI algorithms in this study made FP errors for reasons also shown in previous studies, including normal skeletal growth appearance or growth plate [15], old traumatic findings [19, 24], calcification [24, 25], and bone overlay [24]. Both AI algorithms occasionally missed obvious findings, but most missed findings were classified as subtle, consistent with previous studies [10, 17, 20–22]. The rigorous ground truth criteria applied by two experienced MSK specialists may account for some of the false negatives in our study. In addition, our study showed lower sensitivity but similar specificity values and lower PPV, but higher NPV compared to a previous study including all MSK regions [24]. In contrast to our study, Dell’Aria et al. 2024 included only 101 patients, resulting in a smaller dataset for specific MSK regions, and their study was limited to adult patients [24].

Although numerically somewhat lower, the sensitivity and specificity were not statistically significantly lower in children than in adults for either BoneView or RBfracture. Previous studies in AI’s pediatric interpretation have reported higher [15, 23] or similar [35, 36] values in sensitivity and in specificity [15, 23]. In contrast, Gleamer showed higher specificity values compared to per-patient specificity reported by Franco et al. 2024 [35]. In our study, the upper age limit for the children group was 17 years, whereas in the other study [36], it exceeded 17 years, and our study had fewer patients in the children group [15, 23, 35, 36], which can impact results. In addition, our study showed comparable sensitivity and specificity values for both AI algorithms compared to Oppenheimer et al. 2023 although they only had 31 patients in the children group [17]. Both AI algorithms displayed lower PPV in children than in adults, which may have been caused by the lower prevalence of trauma in children in the study sample.

AI algorithm performance varied between MSK regions. The lowest sensitivity values for BoneView and RBfracture were in the knee interpretation, at 0.760 and 0.640, respectively. This contrasts with previous findings [2, 17], possibly due to a low number of abnormal knee cases in our study. Low sensitivity values and wide variation in knee performance were also reported by a recent meta-analysis [2]. Conversely, the specificity values for BoneView and RBfracture were similar than those previously reported [2, 17]. In the pelvis, both AI algorithms displayed excellent sensitivity values, with higher [17, 33] or similar values compared to the pooled sensitivity in a recent meta-analysis [2, 37, 38] and individual studies [20, 34, 39, 40], while specificities are at similar levels [2, 17, 20, 33, 37–39] or slightly lower than those previously reported [34]. In the pelvis, comparing different studies is challenging, as some have focused on specific findings, such as femoral neck fractures, whereas we included the whole pelvis. Both AI algorithms had comparable sensitivity and specificity levels compared to a recent meta-analysis for ankle and foot [2, 17].

Shoulder interpretation showed similar results to a meta-analysis, where sensitivity and specificity fall within the meta-analytical confidence intervals [2]. In addition, other studies have also demonstrated similar sensitivity and specificity values [17, 25]. For elbow interpretation, our study demonstrated strong performance, with sensitivity and specificity values of 0.959 and 0.924 for BoneView and 0.918 and 0.975 for RBfracture, respectively, compared to previous findings [2, 17]. Relative to previous meta-analytic findings [8, 41], we found similar results in the wrist in sensitivity and specificity values for both AI algorithms. In contrast, previous individual studies showed higher sensitivity values compared to RBfracture [42] or both BoneView and RBfracture [43] but similar specificities [42, 43]. Jacques et al. 2023 demonstrated more modest sensitivity values in wrist interpretation, although the values fall within confidence intervals in our study, and they used CT as the ground truth, which can affect the comparison [44]. In addition, they also included findings in the hand, which makes the comparison difficult. The hand showed higher overall values in our study compared to the wrist, although the CIs overlapped. In our study, wrist had more abnormal than normal findings defined by the ground truth, which can impact the results. In the recent meta-analysis [8, 41], the ground truth varied, including radiologists, orthopedic surgeons, and radiology residents, or original reports checked by a resident with various experience levels, which can also affect the comparison. Furthermore, some studies included only specific fractures, such as distal radius fractures [43], rather than all traumatic findings, which is noteworthy given AI’s limited performance in detecting carpal bone fractures, for example [44, 45]. In our study, many false negatives by the AI algorithms in wrist radiographs were related to carpal instability findings, such as scapholunate interval widening or dorsal intercalated segmental instability (DISI). This suggests that these AI algorithms struggle to detect such subtle findings.

Some limitations of the current study warrant discussion. First, by including all relevant MSK regions, the number of cases in each region was limited. Second, potential bias in the ground truth, defined as the consensus of the subjective views of two experienced MSK specialists, may affect the result. Additionally, we did not re-evaluate the FP findings from AI algorithms, which precludes further insights on the limits of AI performance. Third, AI findings were categorized as normal or abnormal without assessing the size or location of bounding boxes, and the ground truth did not include bounding boxes at the site of findings. Neither did the ground truth include the types of abnormalities, which may limit our results in specific scenarios, such as occult fractures, dislocations, or specific fractures for pediatric patients which are more difficult to interpret in radiographs. Fourth, an AI interpretation was classified as correctly abnormal (TP) even if one of multiple fractures was missed. Fifth, using computed tomography (CT) or magnetic resonance imaging (MRI) as ground truth might be preferred. Sixth, secondary findings, like joint effusion, were not classified in this study, and these cases were classified as normal, in the absence of a fracture or a dislocation.

Based on these results, directions for future studies may be considered. Using cross-sectional imaging (CT or MRI) as the reference standard might provide further insights. Different types of fractures and dislocations should be recorded to give more granular information about AI performance. The sample sizes should be large enough to provide adequate statistical power to assess performance across different MSK regions, specific types of trauma, chronic deformities, and doubtful or borderline cases. Finally, prospective studies on AI deployment in the emergency department would be preferred to gauge additional factors, such as patient and referring physician satisfaction and improvement of workflows.

## Conclusion

In this study, two commercially available AI algorithms, BoneView and RBfracture, showed high and similar diagnostic performance and almost perfect agreement. These findings highlight the potential of AI algorithms in emergency radiograph interpretation.

## Electronic supplementary material

Below is the link to the electronic supplementary material.

Supplementary Material 1

Supplementary Material 2

## Data Availability

Data cannot be publicly shared because of the national legislature on the privacy of patient data.

## Declarations

### AI use

In preparing this manuscript, the authors employed Grok 3 (beta version) and Grammarly Pro to improve the grammatical accuracy and semantic clarity of some sections of the text. The authors then reviewed and revised the content as needed, taking full responsibility for the final published version.
