# Supplementary material for: Comparative accuracy of two commercial AI algorithms for musculoskeletal trauma detection in emergency radiographs
Source: Emerg Radiol. 2025 Jun 9;32(4):569–80. doi: 10.1007/s10140-025-02353-2 (PMC12328546; doi:10.1007/s10140-025-02353-2)
Supplement: Supplementary file 1 — Supplementary Material 1 [file 10140_2025_2353_MOESM1_ESM.docx]

**Comparative Accuracy of Two Commercial AI Algorithms for Musculoskeletal Trauma Detection in Emergency Radiographs**

Emergency Radiology

Jarno T. Huhtanen*, MHSc, PgD^ab^, Mikko Nyman, MD, PhD^c^, Roberto Blanco Sequeiros, MD, PhD^c^, Seppo K. Koskinen, MD, PhD^d^, Tomi K. Pudas, MD^d^, Sami Kajander, MD, PhD^b^, Pekka Niemi, MD, PhD^b^, Hannu J. Aronen, MD, PhD^c^ and Jussi Hirvonen, MD, PhD^ce^

*^a^Faculty of Health and Well-being, Turku University of Applied Sciences; ^b^Department of Radiology, University of Turku; ^c^Department of Radiology, University of Turku and Turku University Hospital, Turku, Finland;* ^d^*Terveystalo Inc, Jaakonkatu 3, Helsinki, Finland; ^e^Department of Radiology, Tampere University, Faculty of Medicine and Health Technology and Tampere University Hospital, Tampere, Finland*

*Corresponding author at: Faculty of Health and Well-being, Turku University of Applied Sciences, Joukahaisenkatu 3, 20520 Turku. E-mail address: [jarno.huhtanen@turkuamk.fi](mailto:jarno.huhtanen@turkuamk.fi)

**Table 3**. Algorithm performance metrics for both AI algorithms in Adults and Children.

|  |  | TP | TN | FP | FN | Sensitivity | Specificity | Cohen’s Kappa |
| --- | --- | --- | --- | --- | --- | --- | --- | --- |
| BoneView (95% CI) | Adults | 320 | 396 | 52 | 35 | 0.901 (0.866–0.928) | 0.884 (0.851–0.910) | 0.81  (0.77–0.85) |
|  | Children | 49 | 122 | 15 | 9 | 0.845 (0.731–0.916) | 0.891 (0.827–0.933) | 0.78  (0.69–0.87) |
| RBfracture (95% CI) | Adults | 312 | 404 | 44 | 43 | 0.879 (0.841–0.909) | 0.902 (0.871–0.926) | - |
|  | Children | 48 | 118 | 19 | 10 | 0.828 (0.711–0.904) | 0.861 (0.794–0.909) | - |

*TP* true positive, *TN* true negative, *FP* false positive, *FN* false negative, *CI* confidence interval
