# Supplementary material for: Comparative accuracy of two commercial AI algorithms for musculoskeletal trauma detection in emergency radiographs
Source: Emerg Radiol. 2025 Jun 9;32(4):569–80. doi: 10.1007/s10140-025-02353-2 (PMC12328546; doi:10.1007/s10140-025-02353-2)
Supplement: Supplementary file 2 — Supplementary Material 2 [file 10140_2025_2353_MOESM2_ESM.docx]

**Comparative Accuracy of Two Commercial AI Algorithms for Musculoskeletal Trauma Detection in Emergency Radiographs**

Emergency Radiology

Jarno T. Huhtanen*, MHSc, PgD^ab^, Mikko Nyman, MD, PhD^c^, Roberto Blanco Sequeiros, MD, PhD^c^, Seppo K. Koskinen, MD, PhD^d^, Tomi K. Pudas, MD^d^, Sami Kajander, MD, PhD^b^, Pekka Niemi, MD, PhD^b^, Hannu J. Aronen, MD, PhD^c^ and Jussi Hirvonen, MD, PhD^ce^

*^a^Faculty of Health and Well-being, Turku University of Applied Sciences; ^b^Department of Radiology, University of Turku; ^c^Department of Radiology, University of Turku and Turku University Hospital, Turku, Finland;* ^d^*Terveystalo Inc, Jaakonkatu 3, Helsinki, Finland; ^e^Department of Radiology, Tampere University, Faculty of Medicine and Health Technology and Tampere University Hospital, Tampere, Finland*

*Corresponding author at: Faculty of Health and Well-being, Turku University of Applied Sciences, Joukahaisenkatu 3, 20520 Turku. E-mail address: [jarno.huhtanen@turkuamk.fi](mailto:jarno.huhtanen@turkuamk.fi)

**Table 4**. Algorithm performance metrics for both AI algorithms in different MSK regions.

|  |  | Hand | Wrist | Elbow | Shoulder | Hip and Pelvis | Knee | Ankle | Foot |
| --- | --- | --- | --- | --- | --- | --- | --- | --- | --- |
| TP | BoneView | 46 | 56 | 47 | 51 | 58 | 19 | 46 | 46 |
|  | RBfracture | 46 | 53 | 45 | 51 | 57 | 16 | 47 | 45 |
| TN | BoneView | 65 | 45 | 73 | 51 | 54 | 98 | 71 | 61 |
|  | RBfracture | 65 | 43 | 77 | 50 | 56 | 96 | 70 | 65 |
| FP | BoneView | 5 | 12 | 6 | 7 | 10 | 4 | 12 | 11 |
|  | RBfracture | 5 | 14 | 2 | 8 | 8 | 6 | 13 | 7 |
| FN | BoneView | 4 | 10 | 2 | 8 | 0 | 6 | 7 | 7 |
|  | RBfracture | 4 | 13 | 4 | 8 | 1 | 9 | 6 | 8 |
| Sensitivity  (Mean, 95% CI) | BoneView | 0.920 (0.812–  0.968) | 0.848  (0.743–  0.916) | 0.959 (0.863–0.989) | 0.864 (0.755–0.930) | 1.000 (0.938–1.000) | 0.760 (0.566–0.885) | 0.868 (0.752–0.935) | 0.868 (0.752–0.935) |
|  | RBfracture | 0.920  (0.812–  0.968) | 0.803  (0.692–  0.881) | 0.918 (0.808–0.968) | 0.864 (0.755–0.930) | 0.983 (0.909–0.997) | 0.640 (0.445–0.798) | 0.887 (0.774–0.947) | 0.849 (0.729–0.921) |
| Specificity  (Mean, 95% CI) | BoneView | 0.929  (0.843–  0.969) | 0.789  (0.667–  0.875) | 0.924 (0.844–0.965) | 0.879 (0.771–0.940) | 0.844 (0.736–0.913) | 0.961 (0.903–0.985) | 0.855 (0.764–0.915) | 0.847 (0.747–0.912) |
|  | RBfracture | 0.929  (0.843–  0.969) | 0.754  (0.629–  0.848) | 0.975 (0.912–0.993) | 0.862  (0.751–0.928) | 0.875 (0.772–0.935) | 0.941 (0.878–0.973) | 0.843 (0.750–0.906) | 0.903 (0.813–0.952) |
| Cohen's Kappa (Mean, 95% CI) |  | 0.86  (0.77–0.95) | 0.72  (0.60–0.84) | 0.87  (0.78–0.96) | 0.71  (0.58–0.84) | 0.85  (0.76–0.94) | 0.76  (0.61–0.91) | 0.79  (0.69–0.89) | 0.79  (0.68–0.90) |

*TP* true positive, *TN* true negative, *FP* false positive, *FN* false negative, *CI* confidence interval
